# New Therapeutic Tools to Shape Monocyte Functional Phenotypes in Leishmaniasis

**DOI:** 10.3389/fimmu.2021.704429

**Published:** 2021-06-25

**Authors:** Natália S. Vellozo, Thaís S. Rigoni, Marcela F. Lopes

**Affiliations:** Instituto de Biofísica Carlos Chagas Filho, Universidade Federal do Rio de Janeiro, Rio de Janeiro, Brazil

**Keywords:** ATRA, *Leishmania major*, M1 and M2 macrophages, monocytes, nitric oxide, RANKL

## Abstract

In the innate immunity to *Leishmania* infection tissue-resident macrophages and inflammatory monocytes accumulate host-cell, effector, and efferocytosis functions. In addition, neutrophils, as host, effector, and apoptotic cells, as well as tissue-resident and monocyte-derived dendritic cells (DCs) imprint innate and adaptive immunity to *Leishmania* parasites. Macrophages develop phenotypes ranging from antimicrobial M1 to parasite-permissive M2, depending on mouse strain, *Leishmania* species, and T-cell cytokines. The Th1 (IFN-γ) and Th2 (IL-4) cytokines, which induce classically-activated (M1) or alternatively-activated (M2) macrophages, underlie resistance versus susceptibility to leishmaniasis. While macrophage phenotypes have been well discussed, new developments addressed the monocyte functional phenotypes in *Leishmania* infection. Here, we will emphasize the role of inflammatory monocytes to access how potential host-directed therapies for leishmaniasis, such as all-*trans*-retinoic acid (ATRA) and the ligand of Receptor Activator of Nuclear Factor-Kappa B (RANKL) might modulate immunity to *Leishmania* infection, by directly targeting monocytes to develop M1 or M2 phenotypes.

## Introduction

Vector-borne *Leishmania* spp. parasites cause leishmaniasis, characterized by localized lesions in the skin or mucosae, as well as disseminated and visceral diseases, which impact health across the globe, particularly in the most impoverished populations. Leishmaniasis affects 12 million people worldwide, while human vaccines are not available and current treatments induce resistance/side effects (1–4). *Leishmania* parasites first infect phagocytes, such as inflammatory neutrophils and tissue-resident macrophages, and then spread to inflammatory monocytes and monocyte-derived macrophages and dendritic cells at the infection site (5–9). The different routes of infection (intraperitoneal, subcutaneous, intradermic, the use of needles or insect bites) underly certain discrepancies in the timing and size of major inflammatory cell subsets (9, 10) and need to be considered when comparing different *Leishmania* experimental models. Undoubtedly, intradermic inoculation mimics better natural infection through sand-fly bites (9).

Environmental stimuli are key to determine macrophage activation and shape macrophage phenotype as permissive host *versus* effector cells during infection. The M1 and M2 terms, coined in reference to Th1 and Th2 responses, describe T-cell independent, strain-specific macrophage features related to the activation of L-arginine metabolism mediated by induced-NO synthase (iNOS) in M1 macrophages or arginase 1 (Arg1) in M2 macrophages (11). The M1 and M2 phenotypes correlate well with classically- and alternatively-activated macrophages, which are induced by IFN-γ (Th1) and IL-4 (Th2) T-cell derived cytokines, respectively (12–17). Most importantly, M1 and Th1 responses predominate in *Leishmania*-resistant strains, whereas M2 and Th2 responses underly susceptibility to infection in specific experimental models (18–23). It is assumed, however, that a range of intermediates from M1 to M2, rather than discrete extreme phenotypes, represents better macrophage plasticity *in vivo* (24, 25).

The role of tissue-resident macrophages and neutrophils in *Leishmania* infection has been addressed at the cutting-edge of knowledge (8, 9, 26, 27). Previous *in vitro* studies showed that neutrophils, either as apoptotic or effector cells, interact with elicited-peritoneal macrophages and affect immunity to *Leishmania major* in a mouse strain-dependent fashion. In resistant C57BL/6 (B6) mice, effector neutrophils helped macrophage activation and parasite killing, whereas in susceptible BALB/c mice, efferocytosis of neutrophils induced *Leishmania*-permissive macrophages (28, 29). Recent image data revealed that dermis-resident macrophages at the skin infection site express M2 phenotype hallmarks, such as the mannose receptor (MR), and are parasite-permissive hosts despite intense Th1 responses to non-healing isolates of *L. major* (8, 26). Remarkably, TAM-mediated efferocytosis of infected neutrophils transferred *Leishmania* parasites to tissue-resident macrophages and reduced innate immunity (9). Similarly, the efferocytosis of infected neutrophils by antigen (Ag)-presenting DCs downmodulated adaptive immunity to *Leishmania* antigens (La-Ag) (6, 30). Therefore, resistance or susceptibility to *Leishmania* infection might not correlate strictly with the Th1/Th2 paradigm.

Here we will discuss the role of inflammatory monocytes in *Leishmania* infection and how we can explore monocyte functional plasticity to shape M1 and M2 responses and promote immunity or restrain exacerbated inflammation.

## Effector Versus Suppressor Monocytes in *Leishmania* Infection

Since early upon *Leishmania* infection, the CCL2-CCR2 axis mediates recruitment of monocytes to infection site where they act as major players in innate immunity to *Leishmania* parasites (31, 32). Mononuclear cells recruited to the skin infection site display either effector or host cell functions depending on resistant or susceptible mouse genotype (33–36). In addition, inflammatory monocytes might also play a suppressive role on anti-parasite T-cell responses (37, 38) in analogy to monocytic myeloid-derived suppressor cells (Mo-MDSCs) that compromise anti-tumoral adaptive immunity (39, 40).

The Mo-MDSC acronym highlights the role of monocytic (Ly6C^+^) cells on T-cell suppression, whereas PMN-MDSC refers to a morphologically and phenotypically (Ly6G^+^) distinct subset (39). Here, we will focus on inflammatory monocytes and Mo-MDSCs from B6 mice, which express equivalent CD11b^+^Gr1(Ly6C)^+^F4/80^int^ phenotypes and morphological aspects, host *Leishmania* parasites, and function as effector cells to control parasite infection (31, 37, 38).

We previously reported that CD11b^+^Gr1^+^F4/80^int^ Mo-MDSCs were present in large proportions in footpad lesions two weeks after *L. major* infection in B6 mice (37). To investigate the role of Mo-MDSCs in *Leishmania* infection, we first injected *L. major* in the peritoneum of B6 mice to generate and sort large numbers of CD11b^+^Gr1^+^F4/80^int^ Mo-MDSCs for *in vitro* and *in vivo* analyses. Purified Mo-MDSCs expressed monocyte morphology and could be promptly infected *in vitro* with *L. major* promastigotes (37). Moreover, Mo-MDSCS controlled infection either spontaneously in a NO-dependent fashion or more efficiently after treatment with T-cell cytokines. Furthermore, co-injection of Mo-MDSCs and *Leishmania* promastigotes in footpads reduced lesions and parasite numbers in draining lymph nodes (LN), concomitant with reduced *ex vivo* T-cell proliferation to La-Ag (37). To directly address suppression of Ag-specific responses, Mo-MDSCs were cocultured with draining-LN T-cells collected two weeks after *L. major* infection. We found that Mo-MDSCs suppressed both polyclonal and Ag-specific T-cell proliferation in a NO-dependent manner (37). Therefore, Mo-MDSCs expressed a NO-producing effector phenotype that might paradoxically suppress subsequent adaptive immunity during *L. major* infection.

Schmid et al. (38) further addressed the role of Mo-MDSC in *L. major* infection by comparing resistant B6 with susceptible BALB/c mice. They showed that BM from B6 mouse yielded increased proportions of Mo-MDSCs compared with BALB/c BM. In addition, in GM-CSF treated cultures, B6 BM produced increased frequencies of Mo-MDSCs, whereas BM from BALB/c mice preferentially generated PMN-MDSCs. Moreover, increased numbers of Mo-MDSCs accumulated in foot pad lesions 10 days after infection in B6 compared with BALB/c mice (38). Similar to peritoneal Mo-MDSCs, Mo-MDSCs from B6 BM suppressed CD4 T-cell proliferation to *L. major* Ag in a NO-dependent mechanism (37, 38). By contrast, Mo-MDSCs from BALB/c BM expressed reduced NO production and are less likely to suppress T-cell responses (38). Therefore, Mo-MDSCs/monocytes play a role as NO-producing effector and suppressor cells in a strain-specific fashion. The observations of increased effector cell responses in inflammatory monocytes from B6 mice might explain, at least in part, innate control of resistance versus susceptibility to *Leishmania* infection in experimental murine models.

## Effector Monocytes and Innate Immunity to *Leishmania* Infection

To investigate the correlation of monocyte functional phenotypes and effective innate immunity, we took advantage of distinct susceptibility versus resistance of BALB/c mice in *L. major versus Leishmania braziliensis* models (41). Early studies suggested that resident peritoneal macrophages infected with *L. major*, but not those infected with *L. braziliensis*, required activation by T-cell cytokines to fight parasite infection (42). Then, we analysed the functional phenotypes of peritoneal exudate cell (PECs) recruited after *Leishmania* infection, by conducting kinetic analyses 1-3 dpi, before the establishment of adaptive immunity (36). We observed that CD11b^+^Ly6C^+^F4/80^int^ monocytes replaced peritoneal-resident (F4/80^hi^) macrophages 1-2 days post infection with either *L. major* or *L. braziliensis*, both in BALB/c and B6 mice. In BALB/c mice, CD11b^+^ PECs developed increased frequencies of M1 hallmarks (IL-12p35^+^ and iNOS^+^ cells), as well as reduced Arg1 expression in response to *L. braziliensis* compared with *L. major* infection. Moreover, restimulation with *L. braziliensis* parasites induced production of proinflammatory cytokines *in vitro*, whereas IL-10 predominated in *L. major-*infected cultures (36).

To address the role of mouse strain, we compared B6 *versus* BALB/c mice infected with *L. braziliensis* and observed that CD11b^+^ PECs from B6 mice expressed increased frequencies of IL-12p35^+^ and iNOS^+^ (M1) cells, but reduced proportions of Arg1^+^ (M2) cells, as well as increased NO production in infected cultures (36). Altogether, monocytes developed M1 responses in a *Leishmania* spp. (*L. braziliensis* > *L. major*)- and mouse genotype (B6>BALB)-dependent fashion. Therefore, the shaping of monocyte phenotypes might be useful to improve M1 responses in therapeutic vaccination of susceptible hosts, based on the benefits of IL-12 administration (43). By contrast, host-directed therapies (2) designed to restrict exacerbated M1 responses to *L. braziliensis* infection might help the control of inflammation (41, 44, 45) in mucocutaneous leishmaniasis.

## Functional Monocyte Responses to CD4 T-Cell Cytokines

When adaptive immunity to *Leishmania* infection ensues, T-cell cytokines IFN-γ and IL-4 play a major role, by inducing classically and alternatively-activated macrophages. Nevertheless, whereas Th1 and Th2 cytokines correlate well with resistance and susceptibility to infection in certain experimental *Leishmania* models, some observations do not fit well with this paradigm and the role of IL-4 is particularly controversial (29). Only recently, Carneiro et al. (46) fully addressed how IFN-γ and IL-4 signalling pathways coordinate inflammatory monocyte responses in *Leishmania amazonensis* infection. Interestingly, although IFN-γ mediated monocyte recruitment to the infection site and induced parasite control by infected monocytes, many other monocytes harboured parasites and sustained infection (46), adding evidence to the notion that monocytes can be parasite-permissive host cells (7, 47). Paradoxically, by downmodulating IFN-γ-mediated recruitment of parasite-permissive monocytes, IL-4 signalling helped resistance rather than susceptibility, particularly in those models where strong Th1 and Th2 responses coexist (46).

In this regard, we previously observed increased Th1 and Th2 responses to *L. major* in vFLIP (caspase-8 inhibitor) transgenic mice, most likely owing to inhibited Fas-mediated apoptosis of CD4 T cells. Moreover, infected B6.vFLIP mice were more resistant to infection than WT mice in an IL-4-dependent fashion (48). Another non-excluding hypothesis to explain IL-4-mediated resistance to *L. major* is the synergism between IL-4 and IFN-γ on macrophage activation for parasite killing (49). Likewise, monocytes from *L. major*-infected B6 mice responded *in vitro* to recombinant IL-4 and IFN-γ *versus* IFN-γ alone, by increasing NO-dependent *L. major* killing (37).

Overall, these studies indicate that monocytes play a key role in innate immunity either as effector cells or permissive hosts for *Leishmania* parasites and that monocyte phenotypes can be modulated by cytokines in adaptive immunity. Therefore, monocytes express functional plasticity and stand as preferential targets for immunomodulation in infectious and inflammatory diseases.

Discrete M1 (IFN-γ/LPS) and M2 (IL-4)-induced macrophages show polarized responses regarding to L-Arginine metabolism (iNOS *versus* Arg1), NO-dependent microbial killing *versus* tissue repair functions, inflammatory cytokines *versus* certain chemokine and surface marker expression (11–14, 17). To address how mouse strain genotype affects monocyte plasticity, we generated BM-derived macrophages (BMDMs) from B6 and BALB/c in the presence of M-CSF. F4/80^int^ BMDMs were then treated with IFN-γ, IL-4, and LPS to provide altogether the environmental correlates of both M1 and M2 conditions. We showed that BMDMs from B6 and BALB/c mice expressed equivalent M1 responses, such as TNF-α and CXCL9 proinflammatory cytokines, as well as NO production in response to mixed M1/M2 conditions (36). Therefore, BMDMs from BALB/c and B6 mice express similar plasticity to respond to Th1 and Th2 cytokines in the infection environment, mimicked by LPS stimulation. It is likely that these experimental settings (M-CSF followed by mixed cytokines and LPS) compensated the BALB/c deficit in the production of BM-derived monocytes (38). We suggest that stronger proinflammatory stimuli, such as LPS plus IFN-γ or *L. braziliensis versus L. major* parasites are less likely to unveil functional differences between BALB/c and B6 monocytes.

In addition to the prototype Th1 and Th2 cytokines, we investigated the role of RANKL, which confers resistance to *L. major* infection in CD40L-defective B6 mice (50). In the B6 model, we cocultured splenic CD4 T cells (from 5 weeks-infected mice) with monocytes (recruited 24 h post i.p. infection), which were then reinfected with *L. major* parasites. We observed that monocytes secreted IL-12 in response to endogenous RANKL and IFN-γ produced during the crosstalk between CD4 T-cells and infected monocytes. In turn, RANKL-stimulated monocytes increased Th1 responses (51). In the next section, we will discuss how RANKL and IFN-γ affect the phenotype of inflammatory monocytes to promote immunity to *L. major* infection (51).

## Targeting Inflammatory Monocytes: RANKL Helps a M2-Like to M1 Phenotype Shift

RANKL (or TRANCE) binds to the Receptor Activator of Nuclear Factor-*Kappa* B (RANK) and activates osteoclasts, macrophages, and DCs (52–54). To address the potential of RANKL to modulate monocyte phenotype, we employed inflammatory peritoneal cells induced upon thioglycolate injection, which expressed RANK. In addition, inflammatory monocytes from B6 mice expressed F4/80^int^ and a M2-like phenotype, characterized by heterogeneous expression of M2 markers such as MGL (CD301), mannose receptor (CD206), IL-4Rα and IL-10 (51). Strikingly, treatment with either RANKL or IFN-γ induced monocytes to express F4/80^hi^ and M1 features, such as IL-12p35 and iNOS expression, IL-12 and TNF-α production, coupled with reduced M2 MGL marker (51). We also observed an intermediate M1-M2 subset, which expressed both MGL and iNOS after treatment with RANKL (51). Likewise, monocytes can co-express iNOS and Arg1, where Arg1 outcompetes iNOS for the L-arginine substrate and promotes *L. major* infection (55). These observations indicate that M2 to M1-phenotypical transition can occur through intermediate phenotypes and it is not an all-or-nothing process.

More importantly, RANKL and suboptimal IFN-γ doses cooperated for the induction of fully-effector M1-macrophages, which showed higher iNOS/NO expression and killed *L. major* parasites in a NO and ROS-dependent fashion (51). Likewise, RANKL and IFN-γ synergized to increase NO production by BALB/c inflammatory monocytes (51). Overall, these findings extend previous observations (50) and indicate a protective role of RANKL in *Leishmania* infection, by inducing M1-mediated immunity (51). Nonetheless, the outcomes of treatment with RANKL *in vivo* depend on the immune context to induce M1 (56) or M2 (57) macrophages. In addition, the systemic therapeutic use of RANKL might affect bone homeostasis, by promoting osteoclast differentiation and activation (54). By contrast, the local uses of RANKL in immunization (58, 59) and in therapeutic vaccines stand as a potential means to improve the activation of DCs and shape macrophage phenotypes in *Leishmania* infection. Interestingly, both RANKL and its inhibitor osteoprotegerin were detected in cutaneous lesions after human infection with *Leishmania tropica* (60).

## ATRA Affects Monocyte-Mediated Immunity to *Leishmania* by Skewing M1 Into M2-Phenotype

All-*trans*-retinoic acid (ATRA), a vitamin A active metabolite that binds to intracellular receptors (61), has been proposed as a potential tool to enhance anti-tumoral immunity, by reducing MDSC-mediated suppression of T cell responses (39, 62). We tested this concept in B6 mice, which were treated with ATRA in the footpads at the time of *L. major* infection. After 17 days, ATRA improved polyclonal T-cell responses concomitant with reduced spontaneous NO production in draining LNs (37). However, treatment with ATRA also increased development of lesions and parasite loads. Moreover, these deleterious effects were recapitulated in peritoneal exudate cells (PECs) collected 24 h after *L. major* infection and then reinfected *in vitro* (37). We observed that treatment with ATRA: (i) increased monocyte differentiation into macrophage; (ii) reduced NO-responses to T cell cytokines; (iii) increased parasite burden in PECs and sorted MDSCs (37). Therefore, ATRA induced monocytes to differentiate into macrophages that were unable to control *L. major* infection, by uncoupling maturation and development of effector functions in monocyte-derived macrophages.

Because treatment with ATRA during infection could have other effects, such as induction of regulatory T cells (61), we further addressed how ATRA affects monocytes, by injecting ATRA i.p. 24 h after *L. major* infection. Increased proportions of F4/80^+^ PECs harboured parasites in the following day after treatment with ATRA, concomitant with reduced detection of inflammatory cytokines in peritoneal exudates from both B6 and BALB/c mice (36). Likewise, reinfection *in vitro* resulted in increased parasite burden in PECs from mice treated with ATRA (36). We concluded that ATRA induced parasite-permissive macrophages regardless of the B6 or BALB/c background. Similarly, vitamin A supplement increased parasite infection in experimental visceral leishmaniasis (63). Conversely, vitamin A might play a protective role in immunity to extracellular parasites (64, 65). Moreover, vitamin A metabolism is part of the alternative macrophage program (65, 66) and contributes to tissue repair (67).

To formally address whether ATRA interferes with the development of M1 and M2 phenotypes, we used the mixed M1/M2 conditions, as described in the previous section (36). Strikingly, BMDMS from both BALB/c and B6 mice developed reduced M1 responses, such as production of CXCL9 and NO, but increased production of M2 chemokines after treatment with ATRA. Moreover, ATRA reduced iNOS expression in F4/80^+^ cells both *in vitro* and *in vivo* (36). Therefore, irrespective of experimental settings and genetic background, treatment with ATRA skewed M1 into M2 or M2-like phenotype, which might be more permissive to intracellular *L. major* parasites.

**Figure 1:**
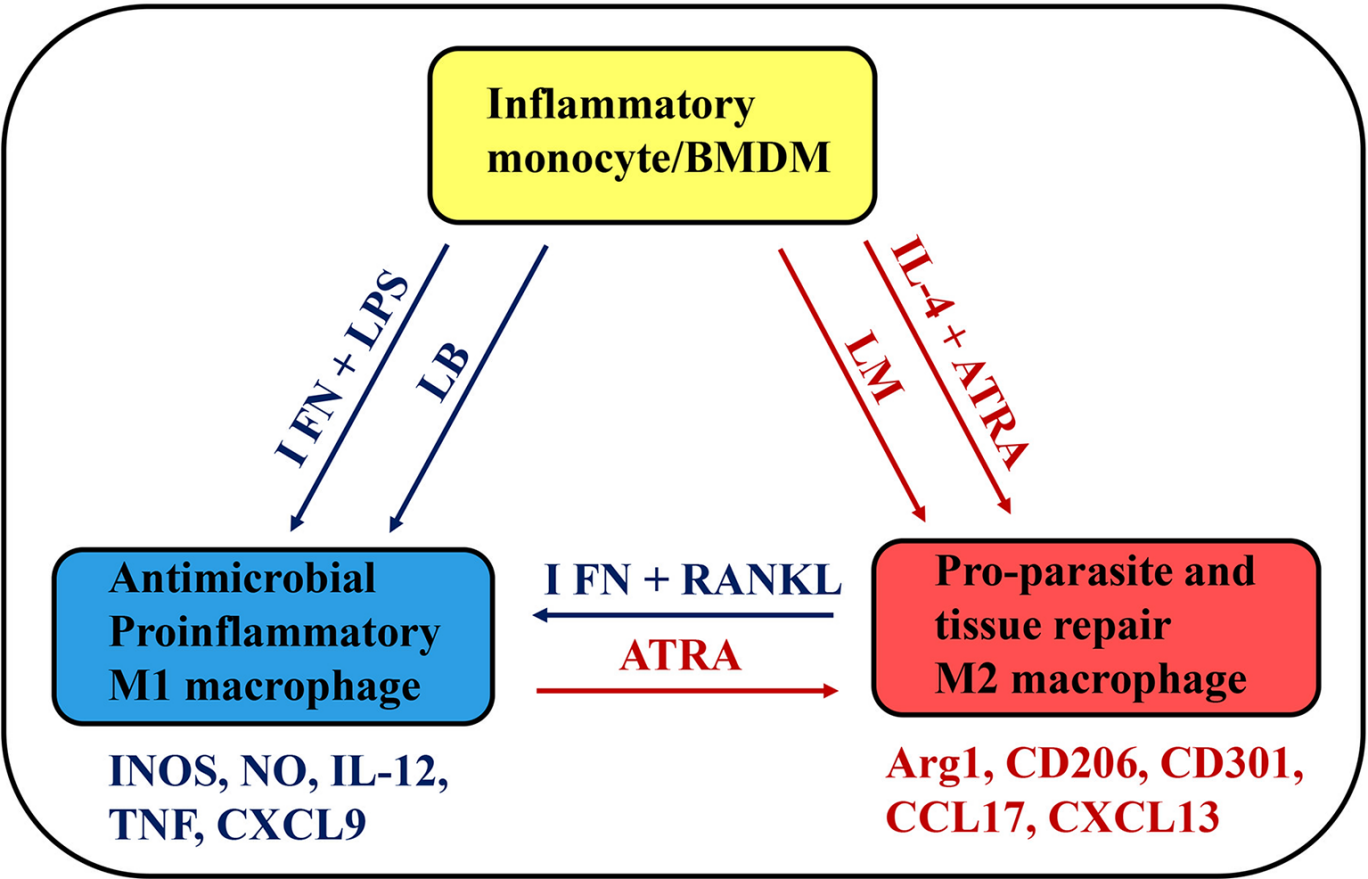
A simplified schematic view of monocyte plasticity as a therapeutic target in *Leishmania* infection. BMDMS and inflammatory monocytes develop into M1, intermediate (not represented) or M2 macrophages depending on environmental stimuli, such as cytokines (IFN-γ or IL-4) and microbial (LPS) or parasite products. *L. braziliensis* (LB) and *L. major* (LM) parasites might differ in their ability to provide stimulus for monocyte activation and differentiation into M1 or M2 macrophages. Potential therapeutic tools, such as RANKL and ATRA have opposing effects on functional phenotypes, by inducing M1 or M2 macrophages, which express a distinct set of skills and play a key role in infection, inflammatory disease, and tissue repair.

More research is needed to address the potential of ATRA for restricting exacerbated macrophage responses to *L. braziliensis* infection as it has been suggested for other inflammatory diseases (68). ATRA reduced inflammatory responses *in vivo*, by limiting NF-κB-activation in macrophages (68). Otherwise, induction of NF-κB signalling pathway underlies increased M1 responses to RANKL plus IFN-γ in inflammatory macrophages (51). Further investigation might formally address the common mechanisms involved in reciprocal M1-M2 phenotype changes induced by ATRA, RANKL (**Figure 1**), and other potential pharmacological tools.

## Conclusion

The targeting of M1-M2 phenotypes includes a range of cell metabolism, signalling, epigenetics, transcriptional and post-transcriptional (i.e. miRNA) (69) targets, which might be able to guide the generation of therapeutic tools able to modulate exacerbated or defective immune responses in inflammation, autoimmunity, cancer, metabolic, and infectious diseases (16, 27, 66, 70–73).

Additionally, the clearance of apoptotic cells changes monocyte phenotype to promote parasite infection (74, 75) and stands as a potential therapeutic target for other diseases as well (76, 77). In a *Leishmania* model, efferocytosis contributes to parasite transfer from apoptotic neutrophils to tissue-resident macrophages and inhibits M1 phenotype to favour infection (9). Accordingly, mouse defective in certain efferocytosis receptors had reduced parasite burden coupled with increased pathology (9). This example highlights the need of accessing the possible outcomes of intervening with the multiple effector and tissue repair macrophage functions to advise caution and prevent potential side effects.

Finally, the use of currently-identified therapeutic tools to modulate monocyte phenotypes demands further research to safely translate preclinical findings into clinical trials and reliable therapies.

## Author Contributions

NV and TR co-wrote the manuscript and contributed equally to this work. ML analysed the literature and wrote the manuscript. All authors contributed to the article and approved the submitted version.

## Funding

This work was supported by the Brazilian National Research Council (Conselho Nacional de Desenvolvimento Científico e Tecnológico, CNPq) and the Rio de Janeiro State Science Foundation (Fundação Carlos Chagas Filho de Amparo à Pesquisa do Estado do Rio de Janeiro, FAPERJ). ML is a research fellow at CNPq, Brazil. We also received fellowships from CNPq (TR), FAPERJ (NV) and the American Association of Immunologists (NV and ML).

## Conflict of Interest

The authors declare that the research was conducted in the absence of any commercial or financial relationships that could be construed as a potential conflict of interest.
